# TPM2 attenuates progression of prostate cancer by blocking PDLIM7-mediated nuclear translocation of YAP1

**DOI:** 10.1186/s13578-023-00993-w

**Published:** 2023-02-23

**Authors:** Zonglong Wu, Liyuan Ge, Lulin Ma, Min Lu, Yimeng Song, Shaohui Deng, Peichen Duan, Tan Du, Yaqian Wu, Zhanyi Zhang, Shudong Zhang

**Affiliations:** 1 Department of Urology, Peking University Third Hospital, Beijing, 100191 People’s Republic of China; 2 Department of Pathology, School of Basic Medical Sciences, Peking University Third Hospital, Peking University Health Science Center, Beijing, China

**Keywords:** Prostate cancer, TPM2, PDLIM7, Hippo/YAP1 signaling pathway

## Abstract

**Background:**

Prostate cancer (PCa) is a common malignant tumor of the genitourinary system. Clinical intervention in advanced PCa remains challenging. Tropomyosins 2 (TPM2) are actin-binding proteins and have been found as a biomarker candidate for certain cancers. However, no studies have explored the role of TPM2 in PCa and its regulatory mechanism.

**Methods:**

TPM2 expression was assessed in Gene Expression Omnibus (GEO) and the Cancer Genome Atlas (TCGA) PCa patient dataset. The effect of TPM2 on PCa progression was assessed in vitro and in vivo by quantifying proliferation, migration, invasion and tumor growth assays, and the mechanism of TPM2 in PCa progression was gradually revealed by Western blotting, immunoprecipitation, and immunofluorescence staining arrays.

**Results:**

TPM2 was found to be severely downregulated in tumor tissues of PCa patients compared with tumor-adjacent normal tissues. In vitro experiments revealed that TPM2 overexpression inhibited PCa cell proliferation, invasion and androgen-independent proliferation. Moreover, TPM2 overexpression inhibited the growth of subcutaneous xenograft tumors in vivo. Mechanistically, this effect was noted to be dependent on PDZ-binding motif of TPM2. TPM2 competed with YAP1 for binding to PDLIM7 through the PDZ-binding motif. The binding of TPM2 to PDLIM7 subsequently inhibited the nuclear transport function of PDLIM7 for YAP1. YAP1 sequestered in the cytoplasm phosphorylated at S127, resulting in its inactivation or degradation which in turn inhibited the expression of YAP1 downstream target genes.

**Conclusions:**

This study investigated the role of TPM2, PDLIM7, and YAP1 in PCa progression and castration resistance. TPM2 attenuates progression of PCa by blocking PDLIM7-mediated nuclear translocation of YAP1. Accordingly, targeting the expression or functional modulation of TPM2, PDLIM7, or YAP1 has the potential to be an effective therapeutic approach to reduce PCa proliferation and prevent the progression of castration-resistant prostate cancer (CRPC).

**Supplementary Information:**

The online version contains supplementary material available at 10.1186/s13578-023-00993-w.

## Background

Prostate cancer (PCa) is one of the most common malignancies of the urinary system. Among men, it is the second most common tumor and the fifth most common cause of tumor-related death [1]. One of the primary treatments for advanced PCa, androgen deprivation therapy (ADT), is known to eventually result in drug resistance. Subsequently, this leads to developing a more aggressive and metastatic disease known as castration-resistant prostate cancer (CRPC) [2]. As CRPC only has a few available therapeutic options, survival prolongation is limited. Therefore, a better understanding of the molecular mechanisms of prostate cancer cells is crucial to developing modern therapeutic tools.

Tropomyosins (TPM) are actin-binding proteins that are expressed in all eukaryotes [3]. Recent studies have highlighted their role in tumor progression [4–6]. Vertebrates have four TPM genes, named TPM1, TPM2, TPM3, and TPM4. The composition and function of the cytoskeleton and focal adhesions involve the mechanical properties of cells that sense and respond to the extracellular environment and influence fundamental cellular processes [7–9]. TPMs are located distal to integrin-based focal adhesions and have been found to play a key role in controlling force generation and stiffness sensing [10, 11]. Recently, a large cohort study has identified TPM2 as a prognostic marker for colorectal cancer. In colorectal cancer cell lines, down-regulated TPM2 has been found to enhance tumor proliferation and migration, whereas TPM2 overexpression attenuates the malignant phenotype of tumor cells [12]. Furthermore, the decreased expression of TPM2 promotes breast cancer metastasis and chemoresistance to paclitaxel treatment and leads to poor survival rates among breast cancer patients [13]. In glioblastoma, TPM2 expression loss induces its spread in brain tissue [14]. However, research studies have yet to explore the role of TPM2 in PCa and its regulatory mechanism.

The Hippo pathway is an evolutionarily conserved tumor suppressor signal transduction pathway, and it ultimately inhibits tumor progression via YAP1/TAZ phosphorylation [15, 16]. Excitingly, many recent observations have suggested a close link between changes mediated by the actin cytoskeleton and the Hippo pathway [17–19]. Hippo signaling comprises a kinase cascade that regulates gene expression for cell proliferation and survival by sensing cell–cell contacts and cytoskeletal information [20–22]. Abnormal Hippo signaling pathways are closely related to the occurrence and development of various tumors [23, 24]. However, the relationship between cytoskeleton-related protein TPM2 and the Hippo pathway remains unclear.

This study explored the functional role of TPM2 in proliferation, invasion, and castration-resistant growth in PCa. We found TPM2 regulates the malignant progression of PCa by blocking the nuclear transport of YAP1. Based on our results, TPM2 regulates the malignant process in PCa cells. Therefore, regulation of TPM2 expression may be a useful strategy in preventing PCa progression and other related therapies.

## Methods

### Identification of different expression genes (DEGs) and hub genes in PCa

The microarray datasets GSE32571, GSE70770, and GSE71016 in Gene Expression Omnibus (GEO) database [25] were used to identify the transcriptomic signature of PCa. GSE32571 dataset contained 39 normal tissues and 59 tumor tissues, while the GSE70770 dataset contained 74 normal tissues and 219 tumor tissues and the GSE71016 dataset contained 47 normal tissues and 48 tumor tissues. Genes with fold change (FC) > 1.5 and P < 0.05 compared with normal tissue in GEO2R tool analysis were defined as PCa-related genes. The overlapping components were analyzed by the Funrich software and visualized by Venn diagrams. The ClusterProfiler software package [26] was used for pathway analysis via Kyoto encyclopedia of genes and genomes (KEGG). PPI network was retrieved from STRING database and reconstructed by Cytoscape software [27]. Cytohubba [28] was used to analyze and select the top 10 hub genes. Next, we obtained the gene expression profile of PCa from TCGA data portal and analyzed it by the Limma package [29].

### PCa specimens and cell culture

After obtaining approval from the Ethics Committee of Peking University Third Hospital, PCa and normal tissue arrays were purchased from Shanghai Outdo Biotech. PCa cell lines C4-2B, PC-3, LNCaP and human embryonic kidney cells (HEK293T) were purchased from the American Type Culture Collection. The normal prostate epithelial cell line RWPE-1 was purchased from Chinese Academy of Sciences cell bank. All cells were cultured in 1640 medium (Biological Industries) containing 10% fetal bovine serum (FBS) (Biological Industries). Cycloheximide (CHX) and MG-132 was obtained from MCE (MedChemExpress). In CHX stimulation experiments, cells were treated with CHX (50 μM) for specific times. To assess the effect of extracellular androgen levels on PCa proliferation, a 10% charcoal-stripped serum-FBS (CSS-FBS) (Biological Industries) medium was utilized. To study enzalutamide resistance, C4-2B cells were cultured in medium containing DMSO or 10 μM enzalutamide (MedChemExpress).

### Establishment of stable cell lines, plasmid construction, and transfection

Human lentivirus-TPM2-overexpressing lentivirus and Flag-YAP1 overexpression plasmids were purchased from Genechem Co. Ltd. Next, Plasmids Flag-TPM2, Myc-TPM2 and Myc-PDLIM7, C-terminal deletion mutant plasmid for TPM2ΔC encoding a TPM2 protein that lacks amino acids 282 to 284, as well as human lentivirus-TPM2ΔC, lentivirus-PDLIM7-overexpressing and the corresponding control lentivirus, were obtained from Youming Biological Technology Co. Ltd. si-TPM2 was purchased from GenePharma Biotech (Shanghai, China) and had the following sequences:

- si-TPM2-1 sense: 5′-CCGACAAGAAGCAAGCUGATT-3′, antisense: 5′-UCAGCUUGCUUCUUGUCGGTT-3′;
- si-TPM2-2 sense: 5′-CUGUGGCAAAGUUGGAGAATT-3′, antisense: 5′-UUCUCCAACUUUGCCACAGTT-3′;
- si-TPM2-3 sense: 5′-GAACUGGACAACGCACUCATT-3′, antisense: 5′-UGAGUGCGUUGUCCAGUUCTT-3′;
- si-N.C sense: 5′-UUCUCCGAACGUGUCACGUTT-3′, antisense: 5′-ACGUGACACGUUCGGAGAATT-3′.

Plasmids or siRNA were transfected using Lipofectamine 2000 (Invitrogen) according to the manufacturer’s protocol. After infection of PCa cells with lentivirus, selection with 4 μg/mL puromycin for 48 h was performed. This was followed by maintenance in a medium containing 2 μg/mL puromycin to obtain stable cell lines.

### Western blot and antibodies

RIPA buffer containing 50 mM tris (pH 7.4), 150 mM NaCl, 1% NP-40, 0.5% sodium deoxycholate and 0.1% SDS was used for cell lysis (Beyotime). Then, 10% sodium dodecyl sulfate-polyacrylamide gel electrophoresis (SDS-PAGE) was to achieve protein separation. Polyvinylidene difluoride (PVDF) membrane (Millipore) was used for protein transfer. After blocking with a 5% blocking solution, the PVDF membrane was incubated with the primary antibody overnight at 4 °C. Following incubation with appropriate secondary antibodies, treatment with Luminata Crescendo Western HRP substrate (Millipore) was performed as well as exposure and digital imaging. The primary antibodies of TPM2 (1:1000, Proteintech, 28587-1-AP), PDLIM7 (1:1000, Proteintech, 10221-1-AP), YAP1 (1:1000, Proteintech, 13584-1-AP), p-YAP1(S127) (1:1000, Abcam, ab76252), NKX3.1 (1:1000, Abcam, ab196020), PSA (1:1000, Abcam, ab76113), c-Myc (1:1000, Cell Signaling Technology, 18583), AREG (1:1000, Proteintech, 16036-1-AP), PCAN (1:1000, Proteintech, 10205-2-AP), HA tag polyclonal antibody (1:1000, Proteintech, 51064-2-AP), GST tag polyclonal antibody (1:1000, Proteintech, 10000-0-AP), MYC tag polyclonal antibody (1:1000, Proteintech, 16286-1-AP) and GAPDH (1:2000, Proteintech, 10494-1-AP). The Beyotime cytoplasmic protein extraction kit was used to extract nuclear proteins based on the manufacturer’s instructions.

### In vivo animal studies

A subcutaneous xenograft tumor model was used to evaluate the effects of TPM2 or PDLIM7 on tumor growth in vivo. The 5 × 10^6^ PC-3 stably overexpressing TPM2 or PDLIM7 cells and their control cells were injected subcutaneously into the armpits of nude mice. After 1 week, the subcutaneous tumor volume was calculated with a digital caliper every 3 days. The subcutaneous tumor volume was calculated according to the following formula: V = width^2^ × length/2. After 3 weeks, the mice were euthanized. The tumor tissues were then weighed and paraffin-embedded for H&E staining and immunofluorescence analysis.

### Immunohistochemistry and immunofluorescence analysis

After deparaffinization and antigen retrieval, the tissue sections were incubated with anti-TPM2 antibody (1:100, Proteintech, 28587-1-AP), anti-YAP1 antibody (1:100, Proteintech, 13584-1-AP), or anti-Ki67 (1:100, Proteintech, 27309-1-AP) antibody overnight at 4 °C. Then, a peroxidase-conjugated secondary antibody was used to detect antigen–antibody complexes. After that, a color reaction was conducted using a 3,3′-diaminobenzidine (DAB) substrate kit (ZsBio). ImageJ software was used for IHC quantification. To perform immunofluorescence staining, PCa cells were seeded on coverslips and fixed in 4% PFA. Cells were then washed and treated with 0.25% Triton X-100 for 15 min. After blocking by 5% donkey serum at room temperature for 30 min, multicolor immunofluorescence staining was performed by Treble-Fluorescence immunohistochemical mouse/rabbit kit (RS0036; Immunoway) according to the manufacturer’s protocol.

### Immunoprecipitation (IP) assay

IP lysis buffer (20 mM Tris–HCl, 150 mM NaCl, and 1% Triton X-100, pH 7.5) was purchased from Beyotime. Cells were lysed in IP lysis buffer. After centrifugation, protein lysis buffer was incubated with antibodies with anti-TPM2, PDLIM7, YAP1, or IgG as control antibodies. This was performed overnight on a rotator at 4 °C, followed by incubation with protein A+G agarose beads (Santa Cruz) for 4 h. The resulting immune complexes were eluted from the agarose beads and analyzed by SDS-PAGE followed by immunoblotting, light chain-specific secondary antibodies (1:2000, CST, 58802S) were used to prevent the IgG heavy chain from obscuring the signal of target protein. The Myc-PDLIM7 and Flag-TPM2 or Flag-TPM2ΔC plasmids were co-transfected into HEK293T cells by Lipofectamine 2000 (Invitrogen). After 48 h, the cells were lysed with IP lysis buffer. Flag fusion proteins were immunoprecipitated by anti-Flag magnetic beads (Beyotime) and eluted with 3X Flag peptide (Beyotime) and analyzed by SDS-PAGE followed by immunoblotting.

### Silver staining and mass spectrometry (MS)

The Flag-TPM2 plasmid was transfected into HEK293T cells. IP lysis buffer was used to prepare the whole cell lysate. The whole cell lysate was immunoprecipitated with Flag magnetic beads or IgG control beads (Beyotime) at 4 °C overnight. The precipitate was washed with cold IP cleaning buffer for 5 times. The eluate was further analyzed by mass spectrometry (MS) and Western blot. According to the manufacturer’s instructions, the rapid silver staining kit (Beyotime) was used to visualize the isolated TPM2 binding proteins.

### Ubiquitination assay

Ubiquitination assay was carried out in HEK293T cells. In HEK293T cells, HA-Ub and Flag-YAP1 were co-transfected with empty vector, Myc-PDLIM7 or Myc-TPM2 plasmids. Cells were treated with 10 mM MG132 for 6 h before harvest. Ubiquitinated YAP1 proteins were enriched by anti-Flag magnetic beads. After eluted with the Flag peptide, ubiquitinated YAP1 was detected by anti-HA antibody.

### GST pull-down assays

Recombinant GST-TPM2 and GST-TPM2ΔC fusion proteins were expressed in bacteria, purified using glutathione-Sepharose and visualized by the Coomassie Blue Staining Kit (epizyme). The Myc-PDLIM7 was expressed in HEK293T cells. Subsequently, the GST-pulldown assays were carried out by incubating GST-TPM2 or GST-TPM2ΔC bound beads with Myc-PDLIM7-expressing HEK293T cell lysates at 4 °C overnight. The beads were washed and the bound proteins were analyzed by Western blot.

### Cell proliferation assay

CCK-8 and 5-Ethynyl-2′-deoxyuridine (EdU) assays were used to assess for changes in cell proliferation. 2 × 10^3^ PCa cells were seeded in 96-well plates and detected by Cell Counting Kit 8 (CCK-8) (Beyotime). EdU kit (Ribobio) was used for EdU determination per manufacturer’s instructions.

### Colony formation assay

PCa cells were seeded in 6-well plates with approximately 1500 cells per well. After 2 weeks of culture, the formed cell clones were stained with crystal violet and counted.

### Transwell migration and invasion assays

100 μL of serum-free medium containing 1 × 10^4^ PCa cells was added to the upper chamber of a transwell migration chamber (8-μm pore size, Costar, New York). 600 µL of medium containing 20% FBS was added to the lower chamber. After 24 h, cells were fixed with 4% PFA and stained with crystal violet. After removing cells on the upper surface using a cotton swab, cells were counted under the microscope in five random fields. For invasion assays, the upper chamber was coated with Matrigel (354480, Corning) before seeding cells.

### Statistical analysis

The data were expressed as means ± standard deviation (SD). Data analysis was conducted using GraphPad Prism software. Statistical analysis was performed using unpaired two-tailed t test, paired t test, Mann–Whitney U tests, two-way ANOVA tests, and one-way analysis of variance (ANOVA) followed by a Tukey’s multiple-comparison. *P* < 0.05 was considered to be statistically significant. The graphical abstract was drawn by *Figdraw software.*

## Results

### Identification of DEGs and hub genes in PCa

**Fig. 1:**
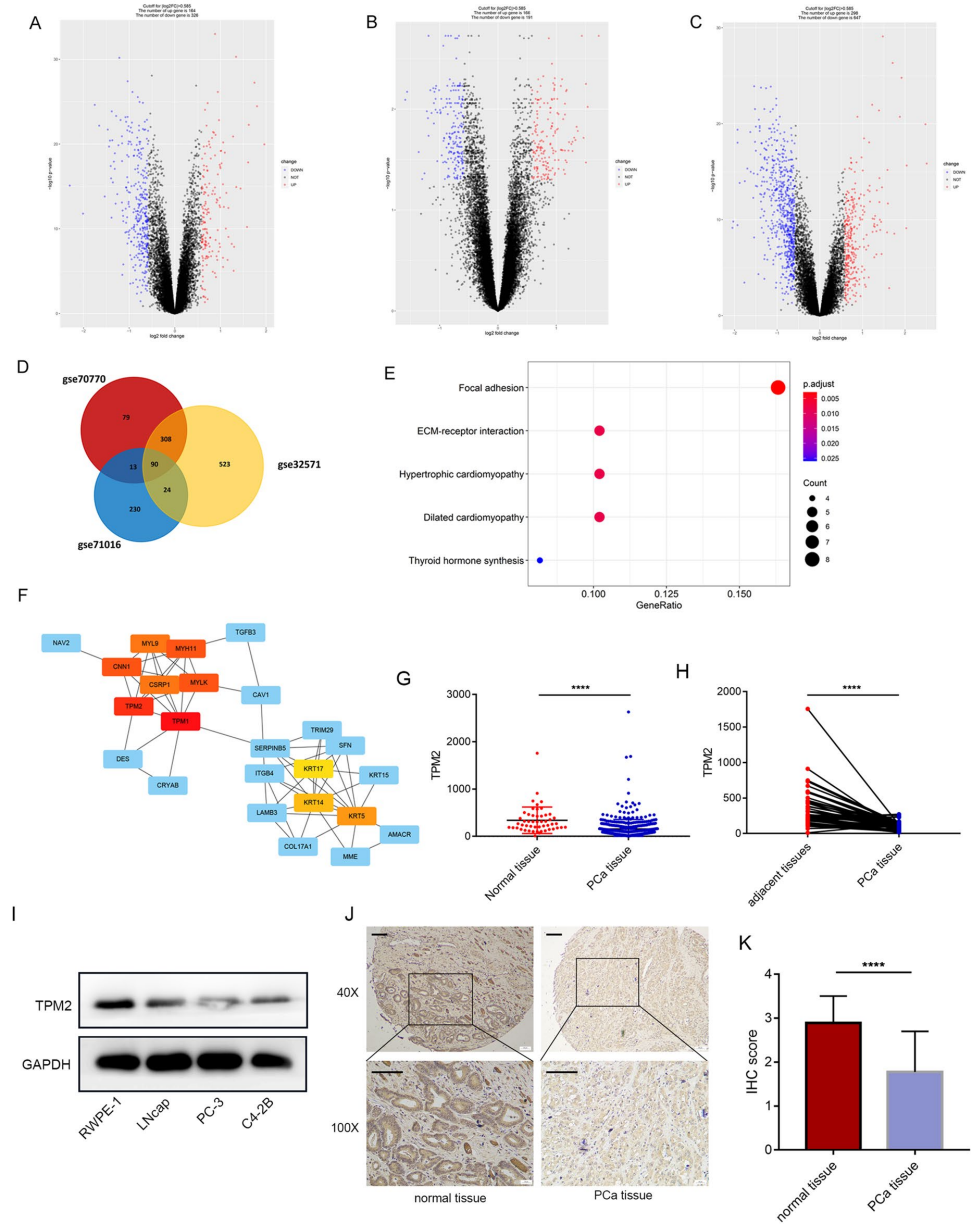
TPM2 expression was downregulated in prostate cancer. **A**–**C** Datasets GSE32571, GSE70770, and GSE71016 were included in the integrative analysis. DEGs filter criteria were fold change > 1.5 and P < 0.05. Volcano plots of DEGs in GSE32571, GSE70770, and GSE71016. **D** Venn diagram depicting common DEGs in GSE32571, GSE70770, and GSE71016. **E** KEGG pathway enrichment analysis. **F** PPI network of identified DEGs and yellow color indicates the hub genes. **G** TPM2 mRNA levels in PCa tissues were assessed from TCGA cohort. **H** mRNA levels in PCa samples compared to paired adjacent normal tissues. **I** The protein expression level of TPM2 was detected in the normal prostate epithelial cell line RWPE-1 and three human PCa cell lines. **J** Representative images of IHC staining of 50 normal prostate tissues and 100 PCa tissues. Scale bars represent 200 μm. **K** IHC score of TPM2 expression levels in normal prostate tissues and PCa tissues. The data were presented as mean ± SD. ****P < 0.0001

To acquire more reliable DEGs in PCa, datasets GSE32571, GSE70770, and GSE71016 were included in the integrative analysis. DEGs filter criteria were fold change > 1.5 and P < 0.05. Based on the results, there were 484 differentially expressed genes (164 upregulated and 320 downregulated genes) in GSE70770, 357 differentially expressed genes (166 upregulated and 191 downregulated genes) in GSE71016, and 945 differentially expressed genes (298 upregulated and 647 downregulated genes) in GSE32571 (Fig. 1A–C). Common DEGs among the three datasets were identified using Venn diagrams, and 90 co-DEGs were identified in PCa (Fig. 1D). To gain an understanding of the co-DEGs in the progression of PCa, KEGG pathway enrichment analysis was used. The results disclosed that these DEGs were significantly associated with focal adhesion and ECM-receptor interaction (Fig. 1E). STRING database and Cytoscape software were used to construct a PPI network. Next, we screened hub gene in DEGs. Hub genes are centrally located inside the module and represent the expression profiles of the entire module, which are often considered as functionally significant and highly connected with other nodes in the module. CytoHubba is a Cytoscape plug-in, which can sort the nodes in the network and distinguish the hub genes in DEGs according to the characteristics of the network [28]. The top 10 hub nodes were identified by cytoHubba, including TPM2, TPM1, MYLK, MYH11, CNN1, CSRP1, MYL9, KRT5, KRT14, and KRT17 (Fig. 1F). As a hub gene, TPM2 may plays an important role in the occurrence and development of PCa. As there is no research to explore the role of TPM2 in PCa. TPM2 was selected to investigate whether its abnormal expression was associated with PCa.

### TPM2 expression was down-regulated in PCa

Next, the dataset from TCGA cohort was used to investigate the changes in TPM2 mRNA levels in PCa tissues. The mRNA level of TPM2 was significantly reduced in PCa tissues (Fig. 1G). Compared to paired adjacent normal tissues, tumor samples had statistically low TPM2 mRNA levels (Fig. 1H). The protein expression level of TPM2 was detected in the normal prostate epithelial cell line RWPE-1 and three human PCa cell lines. As showed in Fig. 1I, TPM2 was decreased in PCa cell (especially in C4-2B and PC3 cell lines) compared with RWPE-1 cells. Next, IHC staining was performed on 50 normal prostate tissues and 100 PCa tissues to assess the clinical significance of TPM2 expression. The staining intensity of TPM2 representative image is shown in Fig. 1J. The expression level of TPM2 decreased significantly in PCa tissue (Fig. 1K).

### TPM2 attenuates the proliferation and invasion of PCa cells

**Fig. 2:**
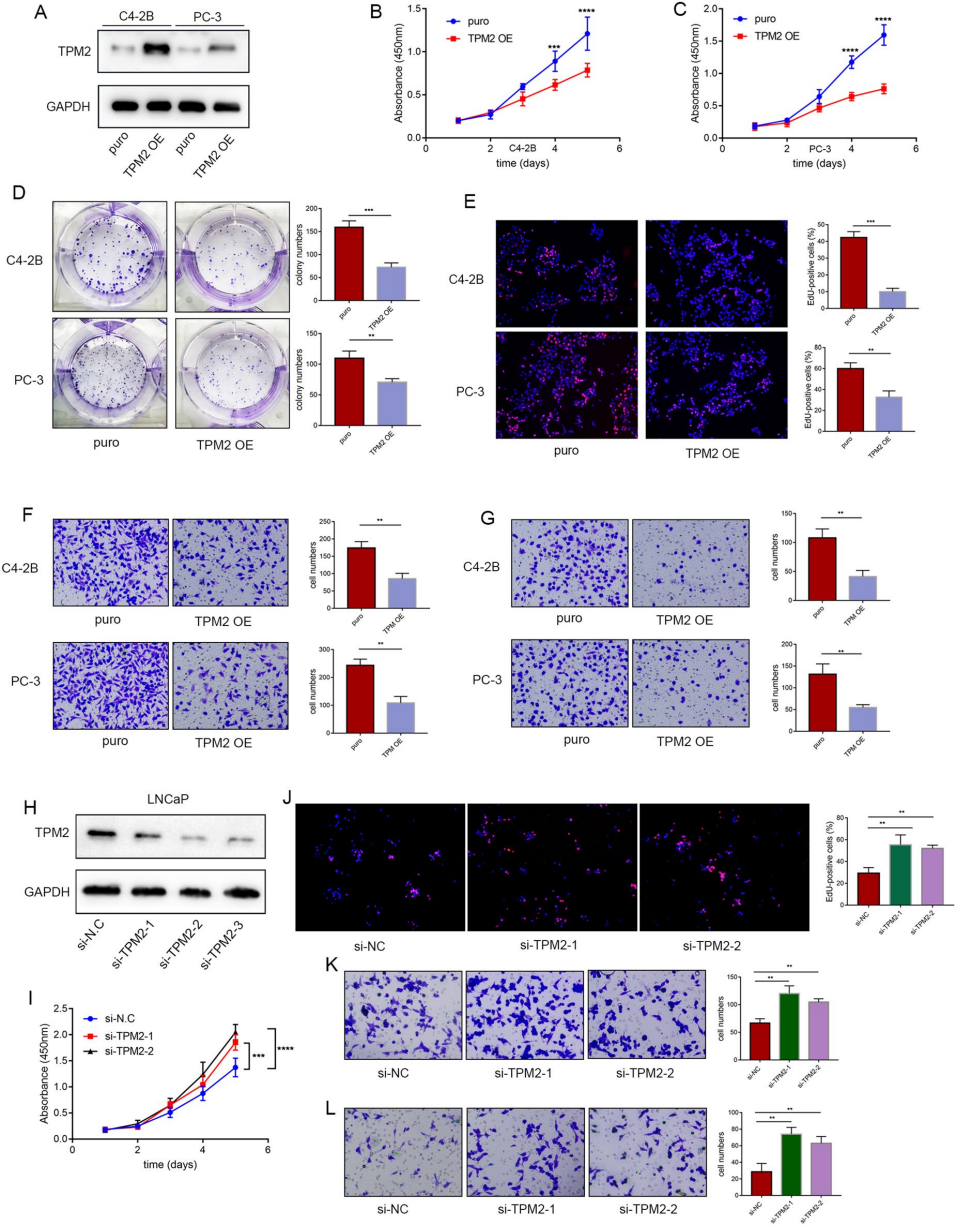
TPM2 attenuates proliferation and invasion of PCa cells. **A**–**G** TPM2 was overexpressed to explore its function in C4-2B and PC-3 cells. The proliferation capacities of the PCa cells were detected by CCK-8, colony formation, and EdU assays. Transwell assays were used to assess the migration and invasion ability of cells: **A** protein expression levels of TPM2 in C4-2B and PC-3 cells following TPM2 overexpression. **B**–**E** Cell proliferation assays: CCK-8, colony formation, and EdU assays. **F**, **G** Transwell migration and invasion assays. H–L The malignant phenotypes of LNcap cells transfected with TPM2 siRNA interference: **H** protein expression levels of TPM2 in LNCaP cell following TPM2 siRNA interference. **I**, **J** Cell proliferation assays: CCK-8 and EdU assays. **K**, **L** Transwell migration and invasion assays. The data were presented as mean ± SD. **P < 0.01, ***P < 0.001, ****P < 0.0001

Next, TPM2 was overexpressed to explore its function in C4-2B and PC-3 cells (Fig. 2A). Using CCK-8 and colony formation assays, TPM2 overexpression was found to significantly reduce cell proliferation and the number of clones. EdU assays further confirmed these results (Fig. 2B–E). Invasion and metastasis are major hallmarks of tumor progression, and overexpression of TPM2 accordingly inhibited the migration and invasion of C4-2B and PC-3 cells (Fig. 2F, G). Additionally, inhibition of TPM2 expression by small interfering RNA in LNCaP cells enhanced its proliferation, migration, and invasion abilities (Fig. 2H–L).

### Low TPM2 expression promotes androgen-independent proliferation of prostate cancer cells

**Fig. 3:**
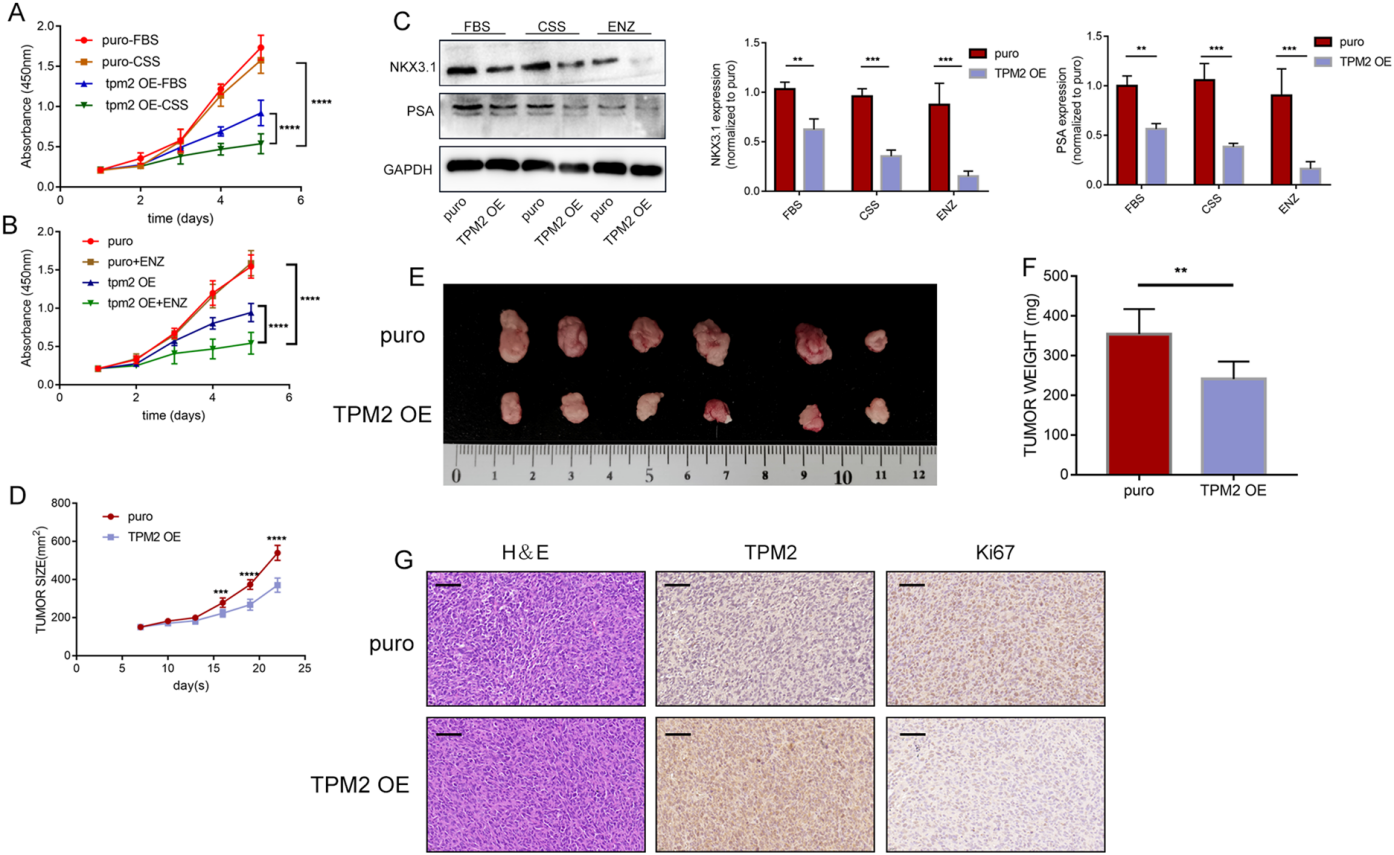
Low expression of TPM2 promotes androgen-independent proliferation of prostate cancer cells. **A** 10% CSS-FBS medium is an androgen-free medium, which was used to explore the effect of TPM2 on androgen level reactivity in C4-2B cells. Proliferation curves for C4-2B cells in CSS-FBS medium. **B** To study enzalutamide resistance, C4-2B cells were cultured in medium containing DMSO or 10 μM enzalutamide. Proliferation curves for C4-2B cells treated with enzalutamide. **C** The expression of AR-dependent genes NKX3.1 and PSA under the condition of 10% CSS-FBS medium and enzalutamide were detected by western blot in C4-2B cells. **D** Tumor growth curves of PC-3 cells express indicated protein in a xenograft mouse model. **E** Images of excised tumors after 3 weeks. **F** Tumor weights. **G** Representative images of the stain of Ki67 and TPM2. Scale bars represent 50 μm. ENZ, enzalutamide. The data were presented as mean ± SD. **P < 0.01, ***P < 0.001, ****P < 0.0001

Androgen is an important physiological medium to promote the progress of prostate cancer by activating androgen receptor (AR). AR has long been the target of prostate cancer. Considering the importance of androgen and AR signal in the development of prostate cancer and the progression of prostate cancer, CSS-FBS medium, which depleted androgen in the medium, was used to explore the effect of TPM2 on androgen level reactivity in PCa cells. In AR positive cells C4-2B, we found that their proliferation was not affected by CSS-FBS medium, however, the inhibition of PCa cell proliferation by TPM2 overexpression was more pronounced under the castrated conditions of CSS medium (Fig. 3A). Enzalutamide is widely used in the treatment of prostate cancer by targeting the ligand binding domain of AR and inhibiting the activity of AR. To investigate whether loss of TPM2 is associated with enzalutamide resistance, TPM2-overexpressing C4-2B cells were treated with enzalutamide. The results displayed that C4-2B were resistant to enzalutamide treatment. However, TPM2 overexpression restored the sensitivity of cells to enzalutamide treatment (Fig. 3B). In AR-positive cells C4-2B, TPM2 overexpression suppressed the expression levels of AR-dependent genes under different experimental conditions, including NKX3.1 and PSA (Fig. 3C). Next, the xenograft model was established. PC-3 cells were inoculated in the armpit of BALB/c nude mice, and TPM2 overexpression was found to significantly delay tumor growth (Fig. 3D). The nude mice were sacrificed 3 weeks later, and the xenografts were dissected and isolated (Fig. 3E). Tumor weights were significantly different between the two groups (Fig. 3F). Moreover, subcutaneous tumor analysis revealed that Ki67 staining abundance was significantly reduced with TPM2 overexpression (Fig. 3G).

### TPM2 interacts with PDLIM7

**Fig. 4:**
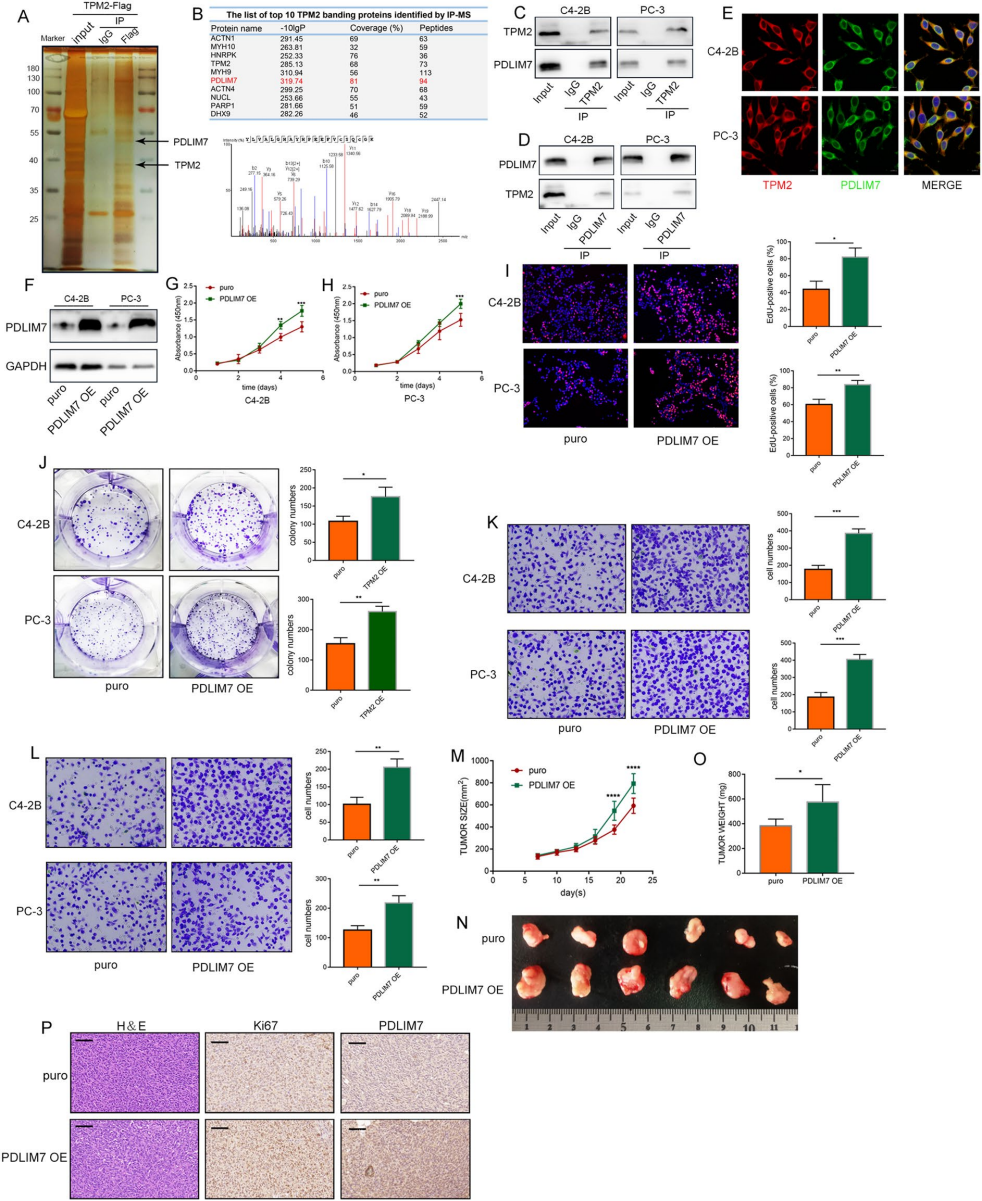
PDLIM-7 promotes the proliferation, migration, and invasion of Pca. **A** Silver-stained gels of immunoprecipitation proteins using anti-IgG and anti-Flag affinity beads to visualize the isolated TPM2 binding proteins. **B** Upper panel: list of candidate proteins from tandem affinity purification-coupled mass spectrometry. Lower panel: MS profiles of PDLIM7. **C** Whole-cell lysates from C4-2B and PC-3 cells immunoprecipitated with TPM2 antibody followed by Western blot analysis. **D** Whole-cell lysates from C4-2B and PC-3 cells immunoprecipitated with PDLIM7 antibody followed by Western blot analysis. **E** Double stains of TPM2 and PDLIM7. **F**–**L** PDLIM7 was overexpressed to explore its function in C4-2B and PC-3 cells. The proliferation capacities of the PCa cells were detected by CCK-8, colony formation, and EdU assays. Transwell assays were used to assess the migration and invasion ability of cells: **F** protein expression levels of PDLIM7 in C4-2B and PC-3 cells following PDLIM7 overexpression. **G**–**J** Cell proliferation assays: CCK-8, colony formation, and EdU assays. **K**, **L** Transwell migration and invasion assays. **M** Tumor growth curves of PC-3 cells express indicated protein in a xenograft mouse model. **N** Images of excised tumors. **O** Tumor weights. **P** Representative images of the stain of Ki67 and PDLIM7. Scale bars represent 50 μm. The data were presented as mean ± SD. *P < 0.05, **P < 0.01, ***P < 0.001, ****P < 0.0001

Next, we explore the mechanism of TPM2 affecting PCa development. MS analysis was used to reveal the proteins that interact with TPM2. MS analysis showed that PDLIM7 protein is a protein that interacts with TPM2 (Fig. 4A, B). As TPM2 contains a PDZ-binding motif at the C terminus, it can potentially interact with PDZ-domain-containing proteins. PDLIM7 was a potential binding partner of TPM2. As expected by MS analysis, IP assays showed TPM2 was interacted with PDLIM7 (Fig. 4C, D). Immunofluorescence staining further confirmed the colocalization of TPM2 and PDLIM7 in PCa cells (Fig. 4E).

### PDLIM7 promotes the proliferation, migration, and invasion of Pca

To further elucidate the role of PDLIM7 in PCa development, a PDLIM7-overexpressing prostate cancer cell line was constructed. PDLIM7 overexpression significantly promotes cell proliferation, migration, and invasion of C4-2B and PC-3 cells (Fig. 4F–L). In addition, the growth of subcutaneous tumors in xenograft mouse model were significantly increased after PDLIM7 overexpression (Fig. 4M–O). Subsequently, Ki67 immunohistochemical staining further confirmed that PDLIM7 overexpression significantly promoted the proliferative ability of prostate cancer cells (Fig. 4P).

### TPM2 blocks PDLIM7-mediated nuclear translocation of YAP1

**Fig. 5:**
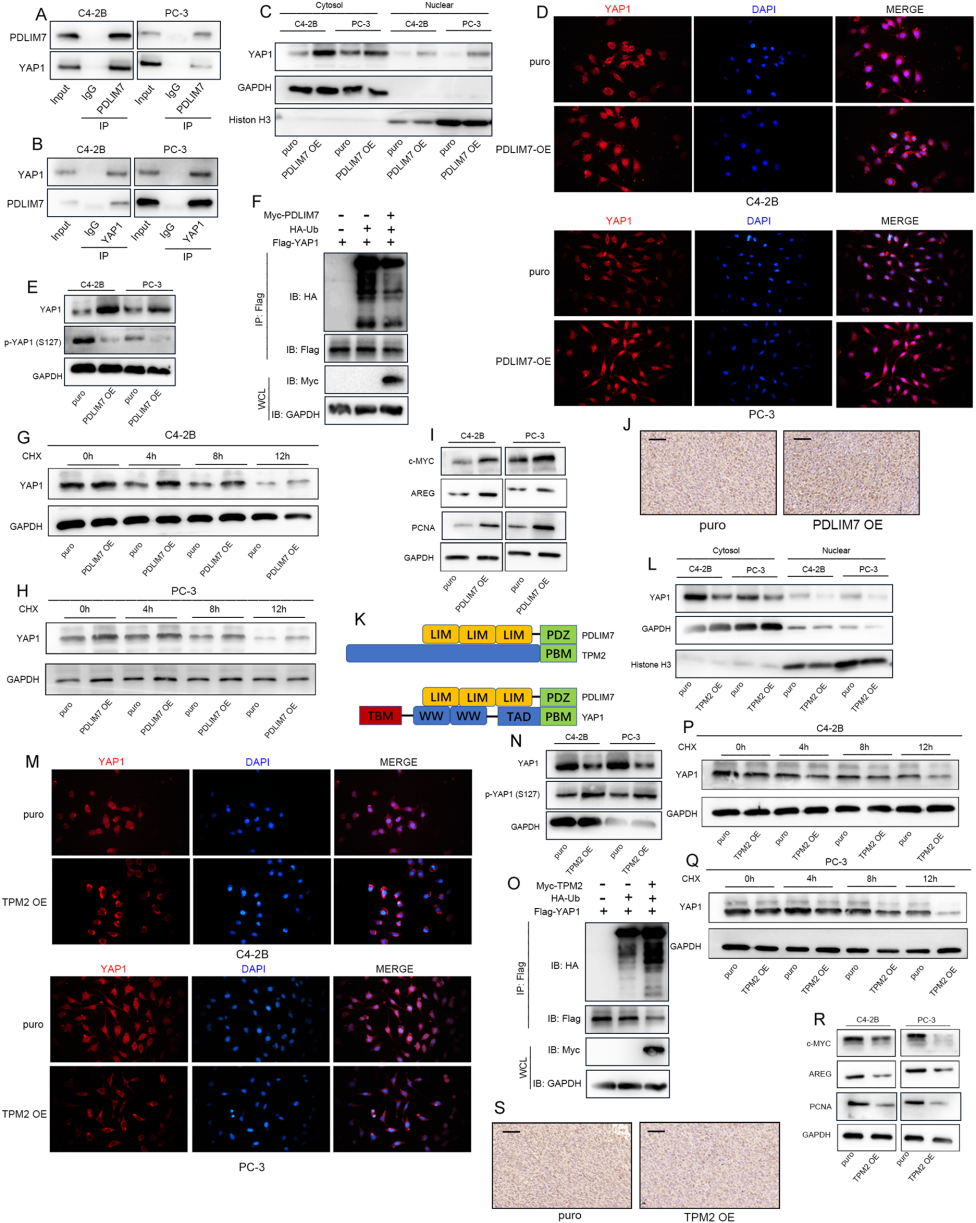
TPM2 blocks PDLIM7-mediated nuclear translocation of YAP1. **A**, **B** Whole-cell lysates from C4-2B and PC-3 cells immunoprecipitated with PDLIM7 or YAP1 antibody followed by Western blot analysis. **C** The level of YAP1 in the nucleus and cytoplasm after PDLIM7 overexpression. **D** Immunofluorescence assay was used to detect the distribution and expression level of YAP1 in C4-2B and PC-3 cells after overexpressing PDLIM7. **E** The expression levels of YAP1 and p-YAP1(S127) in C4-2B and PC-3 cells after overexpression of PDLIM7. **F** HEK293T cells were transfected with HA-Ub and Flag-YAP1 with or without Myc-PDLIM7. After treatment with MG132 (10 µM, 6 h), ubiquitination of YAP1 was measured. **G**, **H** Effect of PDLIM7 on the protein stabilization of YAP-1 in cells treated with 50 µM cycloheximide (CHX) for the indicated times. **I** The expression level of YAP1 target genes: c-MYC, AREG, and PCNA. **J** The stain of YAP1 in PDLIM7-overexpressing subcutaneous tumors. Scale bars represent 50 μm. **K** Protein structure of TPM2, PDLIM7, and YAP1. **L** The level of YAP1 in the nucleus and cytoplasm after TPM2 overexpression. **M** Immunofluorescence assay was used to detect the distribution and expression level of YAP1 in C4-2B and PC-3 cells after overexpressing TPM2. **N** The expression levels of YAP1 and p-YAP1(S127) in C4-2B and PC-3 cells after overexpression of TPM2. **O** HEK293T cells were transfected with HA-Ub and Flag-YAP1 with or without Myc-TPM2. After treatment with MG132 (10 µM, 6 h), ubiquitination of YAP1 was measured. **P**, **Q** Effect of TPM2 on the protein stabilization of YAP-1 in cells treated with 50 µM cycloheximide (CHX) for the indicated times. **R** The expression level of YAP1 target genes: c-MYC, AREG, and PCNA. **S** The stain of YAP1 in TPM2-overexpressing subcutaneous tumors. Scale bars represent 50 μm

Previous studies have mentioned that PDLIM7 can specifically bind to the PDZ-binding motif of YAP1 through a PDZ domain and subsequently promote YAP1 nuclear translocation [30]. Using IP assay, the interaction between PDLIM7 and YAP1 was confirmed (Fig. 5A, B). Next, the cytoplasmic and nuclear proteins were separated, and PDLIM7 overexpression increased YAP1 levels in the nucleus (Fig. 5C). As illustrated in Fig. 5D, immunofluorescence experiments further confirmed that PDLIM7 promoted the nuclear localization of YAP1. Phosphorylation of serine 127 on YAP1 leads to cytoplasmic retention of YAP1, which promotes its ubiquitination, ultimately leading to YAP1 degradation, resulting in down-regulation of YAP1. While non-phosphorylated YAP1 is activated and enters the nucleus to start the transcription of downstream genes [31, 32]. Levels of YAP1 and p-YAP1(S127) were also detected on the Western blot, PDLIM7 decreased the levels of phosphorylated YAP1 at S127 and markedly increased total YAP1 levels (Fig. 5E). Ubiquitination analysis showed that overexpression of PDLIM7 inhibited ubiquitination of YAP1 (Fig. 5F). Therefore, changes in YAP1 protein half-life treated with the protein synthesis inhibitor CHX were measured, and PDLIM7 was found to prolong YAP1 protein half-life (Fig. 5G, H). Next, YAP1 target genes c-MYC, AREG, and PCNA were examined, the results indicated that PDLIM7 promoted the transcription and expression of YAP1 downstream gene (Fig. 5I). Subcutaneous tumor analysis revealed that overexpression of PDLIM7 significantly enhanced YAP1 staining abundance in the nucleus (Fig. 5J).

Since both TPM2 and YAP1 can bind to PDZ domain of PDLIM7, it was speculated that TPM2 might block YAP1 nuclear translocation by competitively binding PDLIM7 (Fig. 5K). Nucleoplasmic protein isolation technique and immunofluorescence experiments confirmed that TPM2 inhibited the nuclear localization of YAP1 (Fig. 5L, M). Additionally, total YAP1 levels were significantly decreased upon TPM2 overexpression, whereas levels of phosphorylated YAP1 at S127 were significantly increased (Fig. 5N). Ubiquitination assay shows that TPM2 promotes the ubiquitination of YAP1 (Fig. 5O). Western blotting after CHX treatment showed that TPM2 facilitated YAP1 degradation (Fig. 5P, Q). TPM2 overexpression inhibited the expression of three YAP1 downstream target genes c-MYC, AREG, and PCNA (Fig. 5R). By subcutaneous tumor analysis, we found that overexpression of TPM2 suppressed the expression level of YAP1, especially the staining abundance in the nucleus (Fig. 5S). Furthermore, we overexpressed TPM2 in breast cancer cell line MCF-7 with endogenous low expression of TPM2. Consistent with the phenomenon found in PCa, nucleoplasmic protein isolation experiments confirmed that TPM2 inhibited the nuclear localization of YAP1 (Additional file 1: Fig. S1A). Additionally, total YAP1 levels were significantly decreased upon TPM2 overexpression, whereas levels of phosphorylated YAP1 at S127 were significantly increased (Additional file 1: Fig. S1B). Therefore, we speculate that TPM2 inhibits tumor development by inhibiting the nuclear localization of YAP1, which is common to other cancer types.

### PDZ-binding motifs are required for TPM2 function

**Fig. 6:**
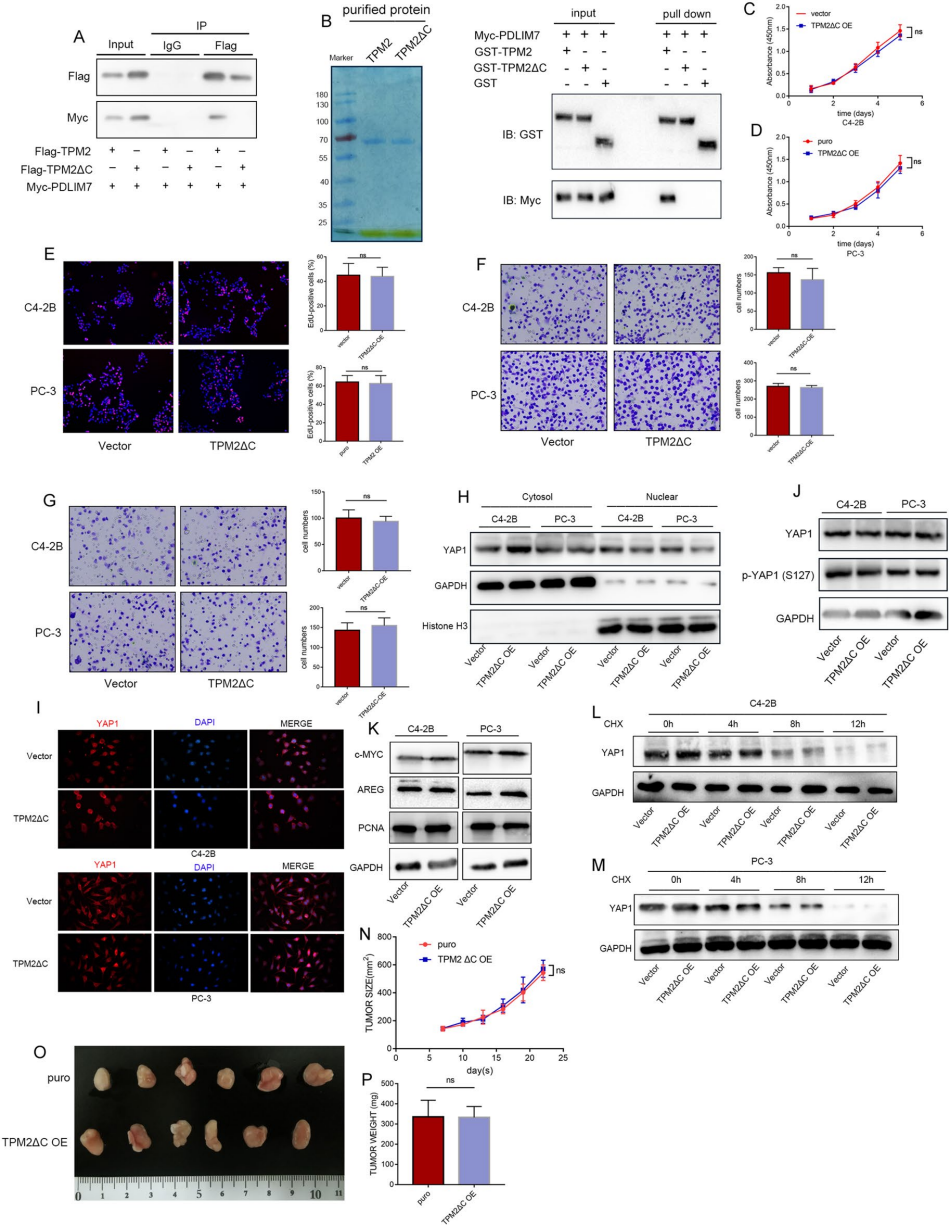
PDZ-binding motifs are required for TPM2 function. **A** Co-IP was performed using cell lysates obtained from HEK293T cells co-transfected with Myc-PDLIM7 and Flag-TPM2 or Flag-TPM2ΔC. **B** Left panel: staining of recombinant GST-TPM2 and GST-TPM2ΔC fusion proteins by Coomassie brilliant blue. Right panel: the GST-pulldown assays were carried out by incubating GST-TPM2 or GST-TPM2ΔC bound beads with Myc-PDLIM7-expressing HEK293T cell lysates and analyzed by Western blot. **C**–**G** TPM2ΔC was overexpressed to explore its function in C4-2B and PC-3 cells. The proliferation capacities of the PCa cells were detected by CCK-8, colony formation, and EdU assays. Transwell assays were used to assess the migration and invasion ability of cells. **C**–**E** Cell proliferation assays: CCK-8 and EdU assays. **F**, **G** Transwell migration and invasion assays. **H** The level of YAP1 in the nucleus and cytoplasm after TPM2ΔC overexpression. **I** Immunofluorescence assay was used to detect the distribution and expression level of YAP1 in C4-2B and PC-3 cells after overexpressing TPM2ΔC. **J** The expression levels of YAP1 and p-YAP1(S127) in C4-2B and PC-3 cells after overexpression of TPM2ΔC. **K** The expression level of YAP1 target genes: c-MYC, AREG, and PCNA. **L**, **M** Effect of TPM2 on the protein stabilization of YAP-1 in cells treated with 50 µM cycloheximide (CHX) for the indicated times. **N** Tumor growth curves of PC-3 cells express indicated protein in a xenograft mouse model. **O** Images of excised tumors. **P** Tumor weights. ns, no significance

Based on the above findings, we speculate that TPM2 competes with YAP1 to bind PDLIM7 through the PDZ binding motif located at its C terminal, which leads to the inactivation and degradation of YAP1. To further investigate if PDZ-binding motifs was required for TPM2 function, a C-terminal deletion mutant plasmid was constructed for TPM2 proteins. HEK293T cells were transfected with Flag‐tagged TPM2 or Flag‐tagged TPM2ΔC and Myc‐tagged PDLIM7. We found C-terminal deletion mutant of TPM2 failed to interact with PDLIM7 (Fig. 6A). GST pull-down assays were performed using recombinant GST-TPM2, GST-TPM2ΔC proteins. The GST pull-down experiments demonstrated a physical interaction of the PDLIM7 with TPM2, but not with TPM2ΔC (Fig. 6B). Next, the effects of transfection with mutant TPM2 on cell proliferation, migration, and invasion in PCa cells were further examined. The TPM2ΔC plasmid transfection was not found to affect the cell proliferation, migration, and invasion (Fig. 6C–G). The function of PBM of TPM2 on the cellular localization of YAP1 was then assessed. TPM2ΔC plasmid transfection did not affect the expression and location of YAP1 (Fig. 6H–J). Furthermore, the loss of PDZ-binding motifs at the C-terminal makes TPM2 lose its role in affecting YAP1 stability and regulating the expression level of c-MYC, PCNA and AREG (Fig. 6K–M). We constructed PC-3 cell lines stably expressing TPM2ΔC. The volume and weight of the tumors in the nude mice xenograft experiment showed TPM2ΔC did not impact the tumor growth (Fig. 6N–P).

### YAP1 overexpression reversed tumor suppression caused by TPM2 overexpression

**Fig. 7:**
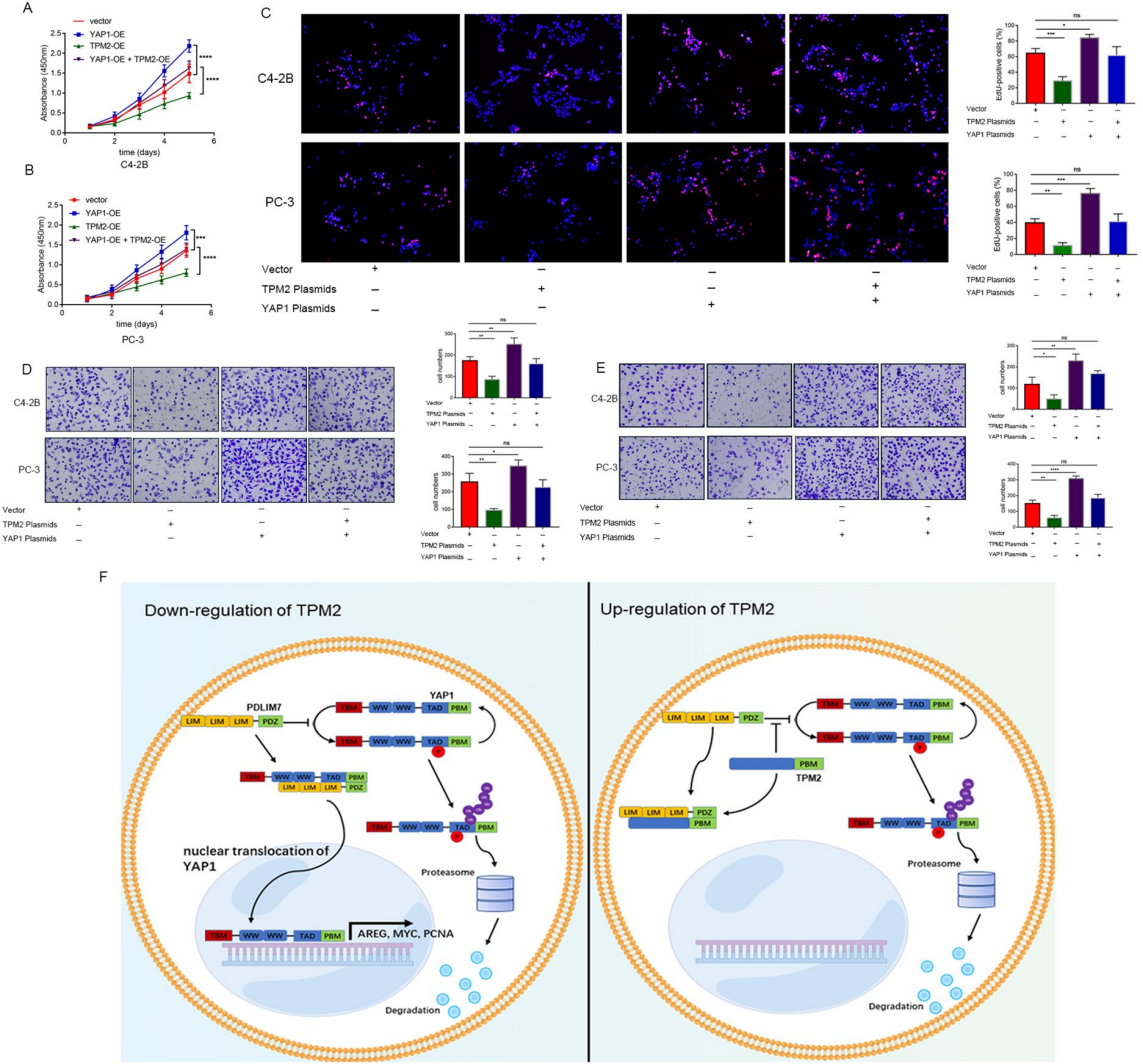
YAP1 overexpression reversed tumor suppression caused by TPM2 overexpression. **A**–**E** The YAP1 overexpression plasmid was co-transfected with TPM2 overexpression plasmid to see if it could reverse the tumor suppression caused by TPM2. The proliferation capacities of the PCa cells were detected by CCK-8, colony formation, and EdU assays. Transwell assays were used to assess the migration and invasion ability of cells. **A**–**C** Cell proliferation assays: CCK-8 and EdU assays. **D**, **E** Transwell migration and invasion assays. **F** Model of TPM2 attenuates prostate cancer progression by blocking PDLIM7-mediated nuclear translocation of YAP1. *PBM* PDZ binding motif, *ns* no significance, *P < 0.05, **P < 0.01, ***P < 0.001, ****P < 0.0001

Next, YAP1 overexpression was tested to see if it could reverse the tumor suppression caused by TPM2. The YAP1 overexpression plasmid was co-transfected with TPM2 overexpression plasmid. It was found that YAP1 overexpression partially reversed the tumor-inhibiting effect of TPM2 overexpression (Fig. 7A–E). Taken together, our findings suggested that TPM2 blocked the PDLIM7-mediated nuclear translocation of YAP1 and YAP1 sequestered in the cytoplasm and underwent further phosphorylation and ubiquitination for degradation, thereby inhibiting the progression of PCa (Fig. 7F).

## Discussion

Despite significant progress in the early detection and treatment of PCa, CRPC still does not have effective treatment options. A better understanding of the molecular mechanisms that control the development and progression of PCa into CRPC will facilitate the research for treatment to prevent PCa progression. In this study, the role of TPM2 in PCa was revealed.TPM2 expression was assessed in GEO and TCGA databases. TPM2 was found to be severely downregulated in PCa tissues. Gain-of-function experiments demonstrated that TPM2 upregulation has a tumor suppressor effect in prostate cancer cells. TPM2 may be a potential candidate for the regulation of PCa initiation and progression. The tumor suppressor effect of TPM2 was associated with blocking YAP1 nuclear translocation and thus leading to its inactivation.

Tropomyosin is a double-chain α-helical coiled-coil actin-binding protein widely expressed in various tissues [33]. Apart from stabilizing the cytoskeleton, TPM also participates in several physiological processes such as cytoplasmic division, cell proliferation, cell migration and motility, apoptosis, and signal transduction [34–36]. Currently, TPM1-4 has been identified as tropomyosin genes in mammals [37]. Several studies have suggested TPM2 as a biomarker candidate for certain cancers. For example, TPM2 expression is downregulated in colorectal cancer and esophageal squamous cell carcinoma [38, 39]. In the present study, the decreased expression of TPM2 was found to promote PCa proliferation, invasion, and castration-resistant growth. Based on MS and IP assays, TPM2 showed interactions with PDLIM7. Immunofluorescence staining further confirmed the colocalization of TPM2 and PDLIM7 in PCa cells. PDLIM7 contains one PDZ domain and three LIM domains [40]. PDZ domain is an evolutionarily conserved domain consisting of 80–90 amino acids, and it mainly binds to the PDZ-binding motif at the C-terminus of proteins [41, 42]. PDZ-binding motifs are divided into three main types: Type I has the S/T-x-I/L/V form, with serine (S) or threonine (T) at − 2 and any amino acids (x) at − 1, 0 for isoleucine (I), leucine (L) or valine (V). Type II motifs have the form Ф-x-Ф, where Ф represents any hydrophobic amino acids. The type III motif is of the form D/E-x-Ф [43, 44]. TPM2 contains a PDZ-binding motif (T-S-L) at the C-terminus, forming the basis for its binding to PDLIM7.

The PDZ binding motif present in the C-terminus of YAP1 is critical for its nuclear localization and functional activation [45]. Based on previous studies, PDLIM7 can specifically bind to the PDZ-binding motif of YAP1 through a PDZ domain and therefore promote YAP1 nuclear translocation [30]. YAP1 is the signaling hub of the Hippo pathway. As a transcriptional cofactor, it forms functional transcriptional activator complexes by binding to transcriptional enhancer-associated domain (TEAD) family genes in the nucleus [46]. YAP1-TEAD complex is involved in the transcriptional regulation of various genes, including genes for cell survival, cell proliferation, cell cycle, cell migration and motility. After the Hippo pathway is activated, YAP1 is phosphorylated and sequestered in the cytoplasm. It is then eventually degraded by the proteasome pathway to remain in an inactive state. Inactivation of the Hippo pathway promotes YAP1 nuclear transfer to initiate transcription of downstream genes [47]. YAP1 shuttles dynamically between the nucleus and the cytoplasm. At high cell densities, YAP1 is mainly distributed in the cytoplasm. However, it can translocate to the nucleus when cells lose contact with each other and/or spread out across their substrate [31]. Importantly, the nuclear localization of YAP relies on the PDZ-binding motif present at the C-terminus [48]. In this study, PDLIM7 was found to bind to YAP1 in PCa, resulting in nuclear translocation of YAP1, thus extending the half-life of YAP1 protein. PDLIM7 overexpression promoted the transcription and expression of YAP1 downstream genes c-MYC, AREG, and PCNA, which in turn promoted the proliferation, migration and invasion of PCa.

Since both TPM2 and YAP1 could bind to the PDZ domain of PDLIM7, it was verified that TPM2 prevented the nuclear translocation of YAP1 by competitively binding to PDLIM7. Importantly, total YAP1 levels were significantly decreased after TPM2 overexpression. TPM2 promoted YAP1 degradation and inhibited the expression of YAP1 downstream target genes c-MYC, AREG, and PCNA. This process relied on the PDZ binding motif at the C-terminus of TPM2. Several studies have found that YAP1 plays a critical role in PCa progression. For instance, YAP1 overexpression in mouse prostate epithelium leads to age-dependent PCa development [49]. Furthermore, in CRPC cells, YAP1 expression is further increased, and high levels of YAP1 expression are critical for CRPC growth and invasion in vitro and in vivo [50, 51]. YAP1 and AR consistently interact in PCa cells for CRPC development, and activation of YAP1 in LNCaP cells leads to androgen-independent growth of LNCaP [52]. Based on these findings, YAP1 plays a key role in PCa by contributing to transition from androgen-dependent growth to castration-resistant growth. Therefore, inhibition of YAP1 signal may be the mechanism of TPM2 regulating the malignant progression of PCa.

## Conclusions

Overall, the results of this study indicate an important regulatory role for TPM2 in PCa development. Specifically, TPM2 overexpression inhibited PCa progression in vivo and in vitro, and this effect was dependent on the interaction of TPM2 with PDLIM7, thereby inhibiting the activation of YAP1 signaling. Therefore, targeting the expression or functional modulation of TPM2, PDLIM7, or YAP1 may serve as a promising therapeutic strategy to reduce PCa proliferation and prevent the progression of CRPC.

## Supplementary Information

**Additional file 1: Figure S1.** TPM2 inhibited the nuclear localization of YAP1 in MCF-7 cells. (A) The level of YAP1 in the nucleus and cytoplasm after TPM2 overexpression in MCF-7 cells. (B) The expression levels of YAP1 and p-YAP1(S127) in MCF-7 cells after overexpression of TPM2.

## Data Availability

The data analyzed in this study were obtained from TCGA data portal and Gene Expression Omnibus (GEO) at GSE32571, GSE70770, and GSE71016. Other data generated in this study were available within the article files.

## Abbreviations

PCa: Prostate cancer
TPM2: Tropomyosins 2
TCGA: The Cancer Genome Atlas
ADT: Androgen deprivation therapy
CRPC: Castration-resistant prostate cancer
GEO: Gene Expression Omnibus
KEGG: Kyoto encyclopedia of genes and genomes
CHX: Cycloheximide
CSS-FBS: Charcoal-stripped serum-FBS
AR: Androgen receptor
DEGs: Different expression genes
MS: Mass spectrometry
