# Supplementary figures and images for: TPM2 attenuates progression of prostate cancer by blocking PDLIM7-mediated nuclear translocation of YAP1
Source: Cell Biosci. 2023 Feb 23;13:39. doi: 10.1186/s13578-023-00993-w (PMC9948342; doi:10.1186/s13578-023-00993-w)

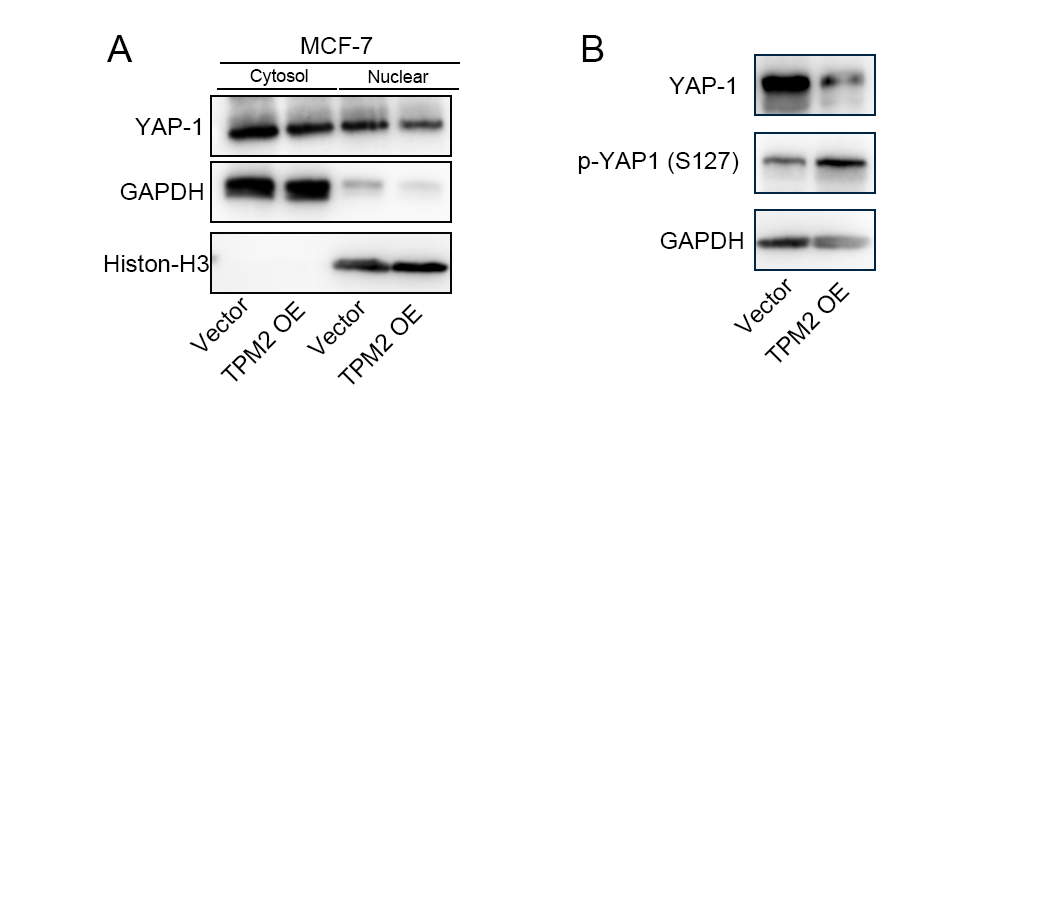

Supplement: Supplementary file 1 — Additional file 1: Figure S1. TPM2 inhibited the nuclear localization of YAP1 in MCF-7 cells. (A) The level of YAP1 in the nucleus and cytoplasm after TPM2 overexpression in MCF-7 cells. (B) The expression levels of YAP1 and p-YAP1(S127) in MCF-7 cells after overexpression of TPM2. [file 13578_2023_993_MOESM1_ESM.tif]
